# Examining the Feasibility of Early Mobilization With Virtual Reality Gaming Using Head-Mounted Display and Adaptive Software With Adolescents in the Pediatric Intensive Care Unit: Case Report

**DOI:** 10.2196/28210

**Published:** 2021-05-27

**Authors:** Byron Lai, Maegen Powell, Anne Grace Clement, Drew Davis, Erin Swanson-Kimani, Leslie Hayes

**Affiliations:** 1 Department of Pediatrics School of Medicine University of Alabama at Birmingham Birmingham, AL United States; 2 Department of Physical and Occupational Therapy Children’s of Alabama Birmingham, AL United States

**Keywords:** physical activity, active video gaming, exergaming, early mobility, rehabilitation

## Abstract

**Background:**

Early rehabilitative mobilization for adolescents is safe and feasible. However, there is a lack of published rehabilitation strategies and treatments that can maximize engagement and outcomes among adolescents in the pediatric intensive care unit (PICU). Virtual reality (VR) gaming using a head-mounted display (HMD) and adaptive software can allow active and nonactive gameplay at the bedside for people with limited arm mobility, making it a potentially inclusive and enjoyable treatment modality for adolescents in the PICU.

**Objective:**

The purpose of this brief case study is to report on the preliminary feasibility of incorporating adaptive VR gaming using an HMD with 2 adolescents who received early mobility treatment within the PICU.

**Methods:**

This study was a mini-ethnographic investigation of 2 adolescents (a 15-year-old male and a 13-year old male) in the PICU who underwent VR gaming sessions as part of their early mobilization care, using an Oculus Rift HMD and adaptive software (WalkinVR) that promoted full gameplay in bed. The Rift was plugged into a gaming laptop that was set up on a table within the patient’s room before each session. The intervention was delivered by an adapted exercise professional and supervised by a physical therapist. Patients had access to a variety of active games (eg, boxing, rhythmic movement to music, and exploratory adventure) and nonactive games (eg, racing and narrative adventure). Gaming sessions were scheduled between usual care, when tolerable and requested by the participant. The interventionist and therapists took audio-recorded and written notes after completing each gaming session. These data were analyzed and presented in a narrative format from the perspective of the research team.

**Results:**

Case 1 participated in 4 gaming sessions, with an average of 18 minutes (SD 11) per session. Case 2 participated in 2 sessions, with an average of 35 minutes (SD 7) per session. Both cases were capable of performing active gaming at a moderate level of exercise intensity, as indicated by their heart rate. However, their health and symptoms fluctuated on a daily basis, which prompted the gameplay of adventure or nonactive games. Gameplay appeared to improve participants’ affect and alertness and motivate them to be more engaged in early mobilization therapy. Gameplay without the WalkinVR software caused several usability issues. There were no serious adverse events, but both cases experienced symptoms based on their condition.

**Conclusions:**

The findings of this study suggest that VR gaming with HMDs and adaptive software is likely a feasible supplement to usual care for adolescents within the PICU, and these findings warrant further investigation. Recommendations for future studies aimed at incorporating VR gaming during early mobilization are presented herein.

## Introduction

### Background

Rehabilitation services for adolescents admitted to a pediatric intensive care unit (PICU), referred to as early mobilization or mobility, have recently been identified as a core component of critical illness management [1,2]. Previously, the general belief system was that adolescents in the PICU were medically unstable and, for safety, should therefore remain immobile and sedated to recover from their ailment [2]. In recent years, this culture has changed rapidly due to randomized controlled trials among adults in the intensive care unit that have demonstrated improvements in pain, delirium, or agitation as a result of proactive treatment [3-7]. As observed in adults, early mobilization in adolescents has also been identified as safe and feasible [2].

Virtual reality (VR) gaming using *off-the-shelf* head-mounted displays (HMDs) is currently the most immersive form of active video gaming that is available to consumers. Owing to recent technological advances, HMDs, such as the Oculus Quest, Oculus Rift, or HTC Vibe, include built-in motion tracking with 6 df to capture bodily movement. Built-in tracking negates the need for externally mounted motion-tracking cameras and, therefore, makes HMDs much easier to deliver across a variety of settings. These HMDs also include high-resolution and framerate displays that provide a seamless gaming experience, which can reduce motion sickness [8,9]. Given that these HMDs are marketed as consumer-available game consoles, they have access to a wide variety of active and nonactive video games in both a single-player and multiplayer web-based format.

Previous studies demonstrated that HMDs can be used in formal rehabilitation to improve motor and executive function, fitness, movement quality, spatial orientation, mobility, and perceived levels of pain [10-13]. Outside of a rehabilitation context, HMDs have also been used to promote serious, health-enhancing exergaming at home among child wheelchair users with a chronic disabling condition [14]. In these applications, the impetus for incorporating HMDs is that most VR games require movements of only the arms for successful play, making it an inclusive and, most importantly, enjoyable treatment modality for adolescents with mobility disability (eg, wheelchair users).

HMDs can be further tailored to adolescents with minimal functional ability with the addition of a commercially available adaptive software, WalkinVR. WalkinVR allows the player or another person (eg, therapist) to adjust the controller, camera, and avatar position during VR gameplay. In addition, the movement of the controllers can be boosted in all directions to project small, real-world movements into large movements within the VR environment. These adaptations make VR gameplay possible from a seated to even supine position and for people with limited arm range of motion. Adaptive VR gameplay holds promise for promoting early mobility at the bedside of adolescents within the PICU, but this has not been investigated.

### Objectives

A few questions must be addressed before designing structured adaptive VR gameplay interventions in the PICU. First, there is a need to identify whether adolescents within the PICU can tolerate active gameplay. Second, if gameplay is tolerable, what duration and intensity of gameplay can they comfortably achieve? Third, is gameplay safe? These questions must be addressed before considering what outcomes could likely be targeted or improved from a gaming intervention in the PICU.

Considering these questions, the purpose of this brief case study is to report on the preliminary feasibility of incorporating adaptive VR gaming using an HMD with 2 adolescents who received early mobility treatment within the PICU.

## Methods

### Overview

A mini-ethnographic case study design was used to explore the feasibility of VR gaming for a convenient sample of 2 adolescents in the PICU of the Children’s Hospital of Alabama [15,16]. A mini-ethnographic case design (ie, focused ethnography) is used to retrospectively develop a rich understanding of the response of an individual or group to a program or study [15]. This approach is smaller in scope and generally shorter in duration than a full-scale ethnographic approach, whereby a researcher is embedded within a setting for a prolonged period to examine the lived experience through more pattern-focused analytical techniques (eg, grounded theory or thematic analysis) [15]. Ethnography is commonly used in medical marketing to investigate the potential of innovative products or programs [15,17].

This study presents quantitative feasibility metrics and supports the quantitative metrics with qualitative narratives.

### Recruitment

This study purposefully selected a convenience sample of 2 adolescents from the PICU at the Children’s Hospital of Alabama. Inclusion criteria were as follows: (1) age ≥13 years (as recommended by the HMD manufacturer), (2) ability to interact with the environment, and (3) limited mobility or conditioning. Exclusion criteria were as follows: (1) substantial visual impairment preventing participation; (2) invasive ventilation or oxygen therapy support, in case they would be compromised when fastening the HMD to the child’s face; and (3) tested positive for COVID-19. This study was conducted in accordance with the case study guidelines set by the Institutional Review Board for Human Use at the University of Alabama at Birmingham. Written informed consent was obtained from a caregiver, with a waiver of assent for the adolescent, before participation.

### Intervention Protocol

Adaptive VR gaming was delivered to participants in addition to their usual care from early mobility physical therapists within the PICU. The early mobility therapy encompassed all typical physical therapy interventions, which were tailored to the patient’s level of alertness, strength, and medical factors. This included range of motion, strengthening exercises, and mobility progression beginning with bed mobility and sitting tolerance up to the point of ambulation and dynamic balance activities. Given the exploratory nature of the study, the gaming intervention was delivered using a learn-as-you-go approach. The frequency of VR gaming sessions was determined by therapists’ judgment on improvement goals for the patient as well as the patient’s willingness to play. The duration and intensity of gameplay during each session was not established a priori, but instead, it was based on how much a patient could comfortably tolerate. Adaptive VR gaming was delivered by the research interventionist (BL), who was a disability exercise specialist, and was supervised by 1 of the 2 early mobilization therapists.

At the start of each session, the research assistant set up the laptop and headset, whereas the therapist prepared the patient for VR. The research assistant placed the laptop on a cooling stand, connected the laptop to the internet on his mobile phone via hotspot, unpackaged the headset and plugged it into the computer, designated the play space within the virtual area (referred to as the *guardian* within the Oculus HMD), opened the Steam and Oculus gaming platform on the laptop, opened and adjusted the WalkinVR software in Steam, fastened the HMD to the head of the patient and the controllers to the hands, and then executed a game on either the Steam or Oculus gaming platform. Participants were instructed to provide a verbal cue when they wanted to switch games or remove the headset. They were instructed to be careful about motion sickness, which can sometimes occur in beginner VR players. After completing the session, the research assistant cleaned and packaged the headset and laptop, whereas the therapist completed the remaining interventions with the patient and provided assistance to return the patient to the starting position.

### Equipment

The patients used an Oculus Rift HMD for VR gaming, which was coupled with 2 handheld controllers. The controllers were equipped with a Velcro strap that was placed over the knuckles (Kiwi design Knuckle Strap). The strap allowed the controllers to be fastened to the hand, thus removing the need to grip and hold onto the controllers during play. The HMD was plugged into a gaming laptop computer (processor: Intel Core i7-10750H, graphics card: NVIDIA, and RAM: 32 GB) on top of a laptop cooling pad, which was positioned on the adjustable tables within the patient’s room. Each day, the research assistant carried the equipment to the patient’s room using 2 shoulder-carried bags, one for the Rift and one for the laptop and cooling stand.

#### Intervention Software

**Figure 1:**
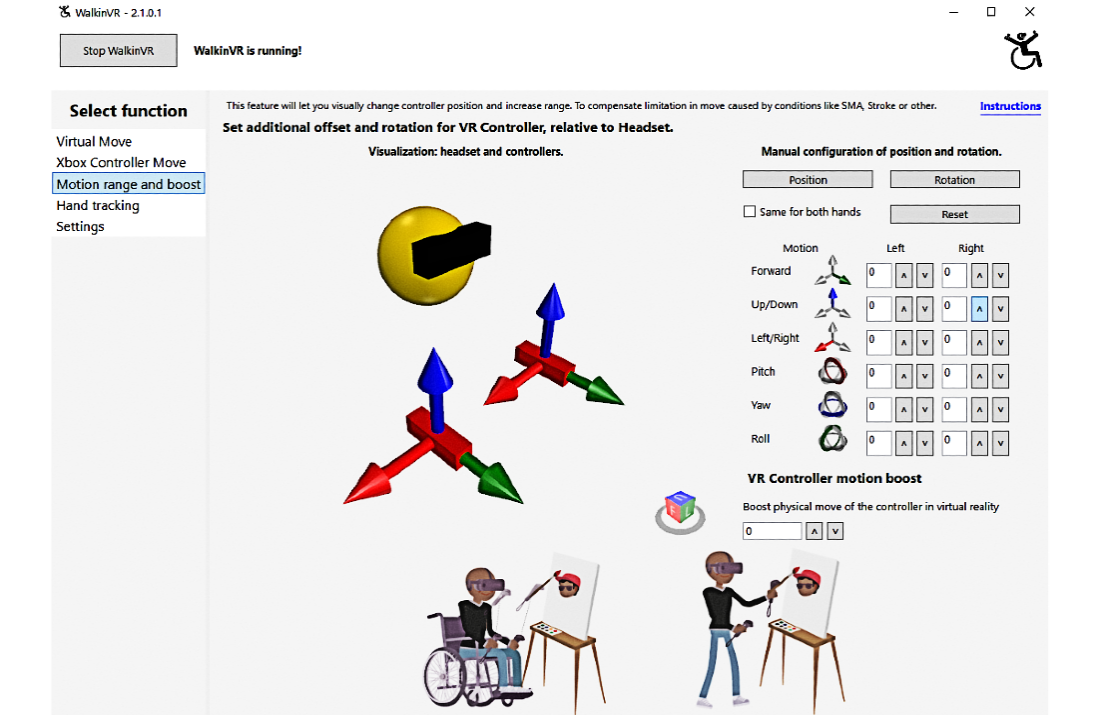
Screenshot of the WalkinVR motion range and boost.

**Figure 2:**
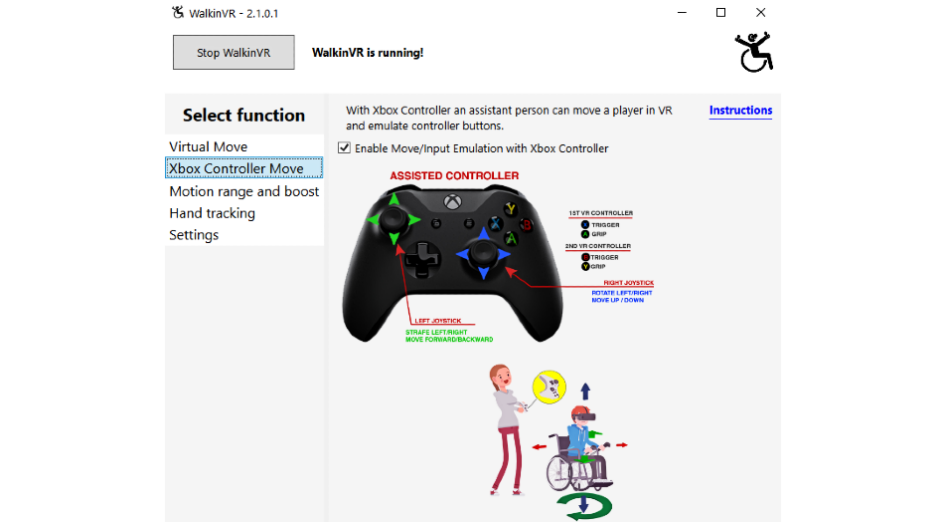
Screenshot of the WalkinVR controller move.

The laptop was installed with software (WalkinVR) [18], which was capable of adapting VR movements for people with disabilities, to enhance the accessibility of gameplay. Specifically, WalkinVR could be set to boost the reach and speed of real-world movements within the VR environment as well as the position of the player’s arms (Figure 1). The software also allows people to play from a range of 0° to 90° (ie, supine to sitting). In addition, the software also allows players to independently rotate and move their in-game character using the handheld controllers to replace the actual locomotion of the player avatar. The player’s avatar could also be controlled externally by another person (eg, trainer or therapist) using an X-box controller (Figure 2). It must be noted that the WalkinVR software operates through the Steam game platform. Thus, only games downloaded from Steam work with the software.

#### Intervention Games

Games for the intervention were installed on the laptop computer, which was connected to the Oculus Rift. The computer was preinstalled with a variety of single-player games to explore participants’ preferences. Games were installed from 2 different internet stores: Steam and Oculus. Games from Steam were active, rhythmic movements to music, sports, and recreation activities that used the WalkinVR software, whereas games purchased from the Oculus store were more narrative and exploratory games that required fewer arm movements. Core games were Beat Saber (movement to music), Thrill of the Fight (boxing), Wolves in the Walls (narrative story), Dirt Rally 2.0 (car racing), Epic Roller Coaster (first-person shooter), and Vader Immortal (adventure). Games were tested before being included in the intervention to ensure that they could be played in a seated position and had high development quality.

### Feasibility Metrics and Case Narrative Description

Feasibility metrics included a variety of exploratory outcomes related to *process, resources, management,* or *scientific outcomes* [19]. The goal of these metrics was to provide a foundation for future trials to design more definitive intervention designs. Process measures included the frequency of gaming sessions and gaming duration in minutes. Resource measures included session setup time (min), session cleanup time (min), the number of staff necessary during the session, and the number of games played by the participants. Management measures included the frequency and type of technical issues experienced by the participants (eg, gameplay issues while seated or at the bedside or computer crashes). Scientific measures included safety, as measured by adverse events. Adverse events were defined by the university’s institutional review board as “...any untoward or unfavorable medical occurrence in a human subject.” An adverse event was recorded and reviewed by the session therapist who classified the event as serious or nonserious and who identified whether it was due to study procedures. Examples of adverse events would include falls, fainting, or nausea.

Narrative case reports were created for each gaming session with each participant. The narrative case reports were based on 2 types of data: (1) the qualitative experience of the research assistant reported via narrative [20] and (2) typed notes from the early mobilization therapists. After each session, the interventionist returned to his office and narratively described his experience of the gaming session. The duration of the audio recordings averaged 25 minutes. While narrating the audio recordings, the interventionist aimed to describe his account of the 4 feasibility metrics that could aid a future pilot trial [19]*.* The audio recordings were transcribed for review purposes. Similarly, therapists created written notes documenting their perspective on the feasibility of VR gaming. Patients provided verbal feedback regarding each game and the overall VR experience, but they were not interested in participating in a formal interview.

### Analysis

This study was formative research aimed at presenting a comprehensive account of feasibility using exploratory quantitative metrics and qualitative case reports, whereby the reader can make their own interpretation of how feasible the intervention can be within their desired context. Accordingly, the outcomes were exploratory, in that there were no a priori criteria that each outcome had to meet to be deemed *acceptable*.

#### Feasibility Metrics

Feasibility outcomes were descriptively reported as means, SD, ranges, and frequencies, when appropriate.

#### Case Report Creation

Qualitative data were synthesized into case reports by the research assistant, who had a background in qualitative inquiry and over 10 years of research experience in exercise training for people with disabilities. Specifically, the research assistant created a narrative story for each gaming session by synthesizing the audio recordings and written notes from the therapists. The therapists reviewed and approved each narrative report. Given that this study was a mini-ethnographic case study, no pattern analyses were used (eg, latent thematic analyses or grounded theory) [15]. The philosophical assumptions that underpinned the data review were ontological subjectivism (multiple realities) and interpretivism (knowledge is created by the interaction of all parties). In other words, the research team acknowledged that the research assistant perceived a reality regarding feasibility, and this reality or experience was recreated by the data provided by the research assistant and therapists.

## Results

### Participant Characteristics

Characteristics of the 2 participants are presented in Table 1.

**Table 1:** Case characteristics.

Characteristic	Value	
	Case 1	Case 2
Age (years)	15	13
Sex	Male	Male
Race	African American	White
Height (m)	1.73	1.70
Weight (kg)	118.8	81.9
BMI (kg/m^2^)	33.9	26.9
Reason for admittance	Rhabdomyolysis (unknown cause)	Severe pancreatitis
Patient notes	Rhabdomyolysis, acute renal failure requiring continuous renal replacement treatment, and hypertensive crisis. Globally weak and deconditioned from muscle damage and prolonged bed rest. Virtual reality intervention began on the 5th day of the patient’s stay in the pediatric intensive care unit.	Hyperosmolar hyperglycemic state, acute respiratory failure requiring intubation, and acute renal failure requiring continuous renal replacement treatment. Globally weak and deconditioned from prolonged sedation and critical illness. Virtual reality intervention began on the 11th day of the patient’s stay in the pediatric intensive care unit.

### Quantitative Feasibility Results

The data for each feasibility outcome are provided in Table 2. In summary, each case completed 2 to 4 sessions of VR gaming in addition to their usual therapy, with an average game time of 27 (SD 14) minutes. Occasionally, other staff (eg, nurses) had to support the research assistant and therapist (described in more detail in the narrative reports). The average number of games played was 6. The average setup and cleanup times were 17 (SD 5) minutes and 8 (SD 3) minutes, respectively. The session setup and cleanup time were slightly increased by the COVID-19 sanitation procedures. Technical issues were not often encountered by the participant, unless the participant was not using the WalkinVR software, which accounted for 80% (8/10) of the technical issues experienced by case 2. Three adverse events were not related to the intervention, as described in detail in the narrative case reports.

**Table 2:** Feasibility results (n=2).

Outcome		Value	
		Case 1	Case 2
**Process metrics**			
	Gaming sessions, n	4	2
	Total gaming duration (min)	70	70
	Gaming duration per session (min), mean (SD; range)	18 (SD 10.6; 6-30)	35 (SD 7.1; 30-40)
**Resource metrics**			
	Session setup time (min), mean (SD; range)	14 (SD 4.6; 10-20)	19 (SD 3.6; 15-23)
	Session cleanup time (min), mean (SD; range)	8 (SD 5.7; 5-12)	8 (SD 2.8; 6-10)
	Staff during session, n (range)	2 (2-3)	2 (2-3)
	Total games played, n	4	6
**Management metrics**			
	Technical issues, n	1	10
	Technical issue type	Game navigation difficulty	Game loading and usability issues without the adaptive software
**Management metrics**			
	Total adverse events, n	1	2
	Nonserious adverse event, n	1	1
	Serious adverse events, n	0	1
	Events related to the project, n	0	0

### Qualitative Case Reports

Narratives for each case are presented below, followed by the recommendations for implementing VR gaming with HMDs in the PICU.

#### Case 1

Case 1 was a 15-year-old African American adolescent boy who was admitted to the hospital for rhabdomyolysis, acute renal failure requiring continuous renal replacement treatment, and hypertensive crisis. Before the first VR gaming session, early mobility therapists noted that the patient was not very responsive to physical therapy. He was asleep or lying down most of the day and did not communicate much with the hospital staff. His therapist noted that he was very interested in trying the VR gaming. His mother stated that he could spend many hours a day playing video games at home, but he had no prior experience playing VR games. His favorite console games were racing or sports games. Case 1 completed 4 video gaming sessions.

In session 1 (October 26, 2020), he completed 22 minutes of play. He had the most success and spent the most time playing a semiactive narrative game (Wolves in the Walls). He was very focused and engaged with the game and completed an entire chapter of the story within the game. The game required minor head or neck movements and active reaching and chopping movements of the arms. He tried a more active game, Beat Saber, using the WalkinVR software. The interventionist boosted case 1’s arm movements and increased the reach using the WalkinVR software, which allowed case 1 to successfully chop the boxes, but case 1 had difficulty keeping up with the rhythmic arm movements to successfully chop the boxes in the virtual world. The therapist reported that difficulty chopping was likely due to arm pain caused by rhabdomyolysis. At the end of session 1, case 1 requested to play boxing or racing for his next session. The hospital staff were amazed at how active and engaged case 1 was during the session. The nurse and therapist noted that this was the most awake they had ever seen him, as he slept most of the day. The interventionist took 20 minutes to set up the laptop and HMD and obtain written consent. The therapist and nurse raised case 1’s bed to an upright position (approximately 75°). The therapist performed manual stretching with the patient while waiting for the VR setup.

**Figure 3:**
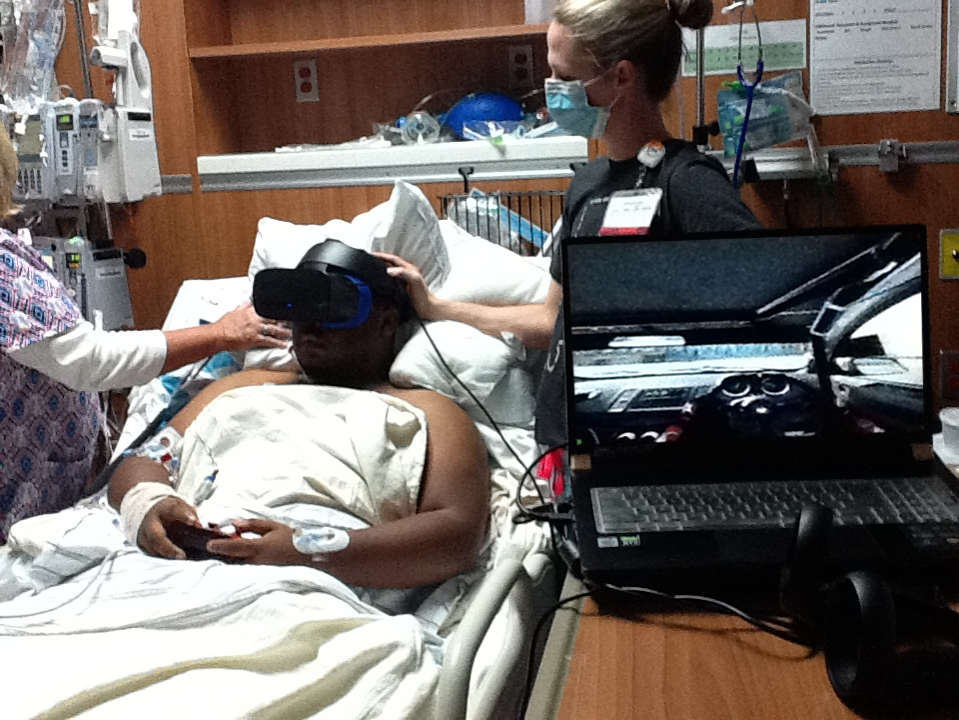
Demonstration of virtual reality racing in the pediatric intensive care unit.

In session 2 (October 27, 2020), case 1 completed 12 minutes of play. Case 1 was woken from his sleep, and it took 15 minutes for him to be prepared in the upright position and be mentally awake for gameplay. The patient was noticeably more fatigued than in the previous session. Case 1’s dialysis access had changed between sessions 1 and 2, and his vascular catheter was now in his right internal jugular vein, which caused him pain when he tried to turn his head. The therapist physically held the line throughout the session to avoid pain or interference during case 1’s gameplay. The interventionist took 11 minutes to complete the setup. Case 1 completed 12 minutes of racing gameplay (Dirt Rally 2.0) using an X-box controller instead of the motion-tracking controllers (Figure 3). He reported that he played the same game on his gaming console at home and was thus heavily engaged in the game after putting on the headset. He navigated the game menus and drove the cars with a high level of skill. He appeared comfortable during the 12 minutes of gameplay, so much so that he did not say a word during gameplay until the end. Near the end of the play, he reported that his stomach felt hot. The nurse and therapist positioned a portable fan near the bedside, which made him feel better temporarily. He chose to stop gameplay after 12 minutes because of this issue. The PICU staff concluded that the issue was because he did not eat or drink anything throughout the day. He reported that he wanted to sleep. The therapist lowered the patient in his bed to a near-supine position. He went back to sleep shortly after the interventionist and therapist exited the room. Overall, amidst these challenges, the PICU staff reported that case 1 participated well, and the gaming appeared to benefit his affect more than any sort of physical function (due to the dialysis circuit preventing him from any significant upper extremity movement).

**Figure 4:**
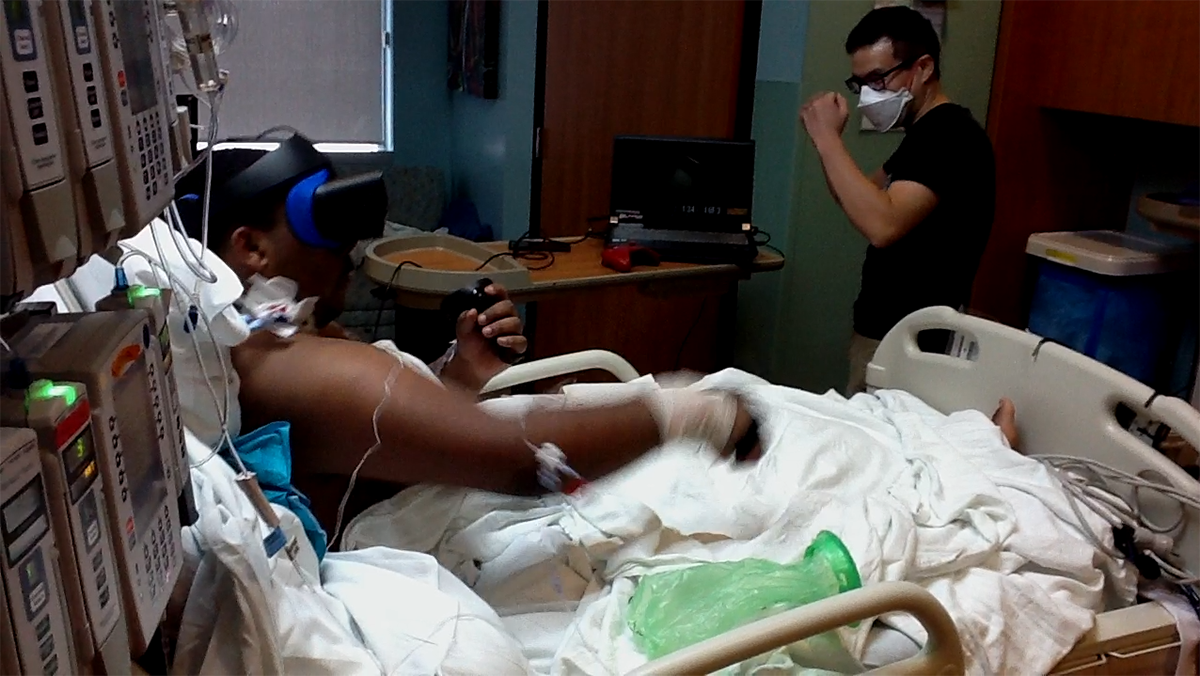
Demonstration of virtual reality boxing in the pediatric intensive care unit.

In session 3 (October 28, 2020), case 1 completed 6 minutes of moderate-to-vigorous intensity exercise in a boxing game (Thrill of the Fight) using WalkinVR. Before starting the session, the therapist found that case 1’s foot was pressed against the bedframe and resolved the issue before play. Using WalkinVR, the interventionist provided case 1 with physical boost to his arm movements, adjusted the player height and arm positions, and moved the player avatar with an X-box controller during gameplay. The interventionist also moved case 1’s player avatar to complete the initial setup within the game. The game asks the player to stand up on a virtual scale to calibrate the player height, which was not necessary for the intervention because the player height could be adjusted within WalkinVR. This setup took 15 minutes. Case 1 then proceeded to complete a 3-minute round of seated boxing against a virtual heavy bag and training dummy, which was followed by one 3-minute round of boxing against a computer opponent (Figure 4; Multimedia Appendix 1). He appeared very focused on the gameplay and was throwing flurries of straight and hook punches. Case 1 achieved substantial reach on his punches, which was amazing as none of the hospital staff had seen case 1 as active as he was while boxing. The interventionist, being an ex-amateur boxer, prompted case 1 to remember to breathe while boxing and throw boxing punch combinations (eg, jab, cross; and jab, jab, cross). The interventionist and case 1 worked together as a team to beat the computer opponent. Unfortunately, the session was interrupted because case 1 had to be moved to another room and undergo an x-ray and other medical procedures. It must be noted that case 1’s punching would sometimes cause his catheter to get caught on the bedside, which required constant monitoring by the therapist.

In session 4 (November 3, 2020), case 1 completed 30 minutes of play. Case 1 had not undergone a VR session for several days because of pain due to rhabdomyolysis. Case 1 was feeling much better than his last session. He was ambulatory, conversive, and had a positive affect. He was transferred to an acute care floor of the hospital. He chose to play the games in a lounge area of the hospital floor and was connected to an intravenous drip during play. Setup took 10 minutes. Case 1 played Thrill of the Fight and Beat Saber. He played 2 rounds of boxing and required very little physical boost in WalkinVR. He played 3 rounds of Beat Saber, with high success in box chopping. He tried the easy and hardest level of difficulty and was highly engaged in the play. Case 1’s movements were very dynamic and near full range of motion. He reported that he was playing at a moderate intensity, as indicated by his breathing rate and a verbal rating of perceived exertion of 6 on a 1-10 scale. Overall, case 1 was exercising for nearly the entire 30-minute session. He was willing to play longer but had another medical appointment that he needed to attend.

In summary, case 1 had a linear progression in the game genre from nonactive exploratory games to active exergaming. This progression was achieved across a small number of sessions. Adaptations from the WalkinVR software were heavily relied upon during the earlier gaming sessions but could be gradually reduced over time to accommodate patient progression. The interventionist acted as the gaming instructor and handled several technical nuances during gameplay (eg, setting up the play area, equipping the headset to the participant, managing adaptations to gameplay in WalkinVR, and coaching the patient on game mechanics). The therapist ensured that the gameplay was safe and comfortable for the participant. The therapist briefed the interventionist on the patient’s medical status before each session. No serious adverse events occurred during gaming using the HMD. Following completion of VR treatment, case 1 remained hospitalized for 2 more weeks for the management of renal and nutritional issues. He participated well in therapy throughout this time and was motivated to improve. When he left the hospital, he was able to walk >1000 feet independently with no shortness of breath or loss of balance and appeared to be close to his baseline level of function.

#### Case 2

Case 2 was a 13-year-old White adolescent boy who was admitted to the hospital for severe pancreatitis, hyperosmolar hyperglycemic state, acute respiratory failure requiring intubation, and acute renal failure requiring continuous renal replacement therapy. Case 2 had global weakness and poor cardiovascular endurance after 7 days of mechanical ventilation and 4 days of paralytics due to critical illness. The therapist reported that initially the patient was shy and difficult to converse with. Similar to case 1, case 2 was asleep or lying down most of the day and was excited to try VR gaming. He reported that he was familiar with video games, knew about the headset, and watched demonstrations of VR gaming on YouTube. Case 2 completed 2 sessions.

In session 1 (November 2, 2020), case 2 completed 40 minutes of play in bed. Case 2 had an additional 15-minute physical therapy session earlier in the day, focusing on traditional strengthening exercises. The setup took 15 minutes, which included obtaining written consent. Case 2 started playing Beat Saber and was highly engaged during play. He voluntarily transitioned from supported sitting with the head of the bed elevated to 75° to unsupported upright sitting and leaning forward during gameplay. Similar to case 1, case 2’s real-world movements were small and dependent on physical boosts and the extended reach provided by the WalkinVR software. He chopped the boxes with moderate success during gameplay. After completing 3 songs on Beat Saber, case 2 asked to try as many games as possible. He played Dirt Rally 2.0 using the X-box controller. When he was handed the controller, he had a noticeable familiarity with it. He demonstrated a high level of skill in racing a virtual car. He completed 3 levels in Angry Birds, which requires handheld controller movements that replicate shooting a slingshot. He played 2 rounds of boxing (Thrill of the Fight) and had a high level of engagement. He punched sporadically throughout the rounds and appeared fascinated by the immersion within the virtual environment (he looked at his surroundings and the realness or interactive quality of his virtual gloves). Case 2 and the interventionist tried a few other games, but some games would not load on the laptop (likely because of poor internet connectivity). Case 2’s heart rate was around 130 beats per minute, with an oxygen saturation level between 99% and 100% during gameplay. At the end of the session, case 2 reported that he enjoyed the gameplay and was noticeably more conversive and alert than he was before the session. The therapist noted that he was more interested in VR activities than therapeutic exercises. He appeared physically tired at the end of the session, and he laid back in bed after the session. It appeared that completing the session motivated the participant to become more active in the real world. He was able to take off the headset himself. Case 2’s mom stated that the VR session was the most awake and active she had seen him in the hospital. Case 2 did not report any pain, nausea, or dizziness.

In session 2 (November 3, 2020), case 2 tolerated 50 minutes of out-of-bed activity and 30 minutes of play. The setup for gameplay took 23 minutes, as the patient was transferred from a bed to a chair. The patient was asked to rest quietly before starting the session. The therapist noted that it would be beneficial for the patient to get out of the bed and transfer into the bedside chair to progress his functional mobility and then remain seated in the chair for play. The patient was carefully transferred to the chair with the assistance of the therapist, and he played all games in a seated position. He maintained an upright position with the trunk unsupported, while being seated on the edge of the chair throughout gameplay and using abdominal musculature to maintain seated balance. The therapist and interventionist supervised him and verbally cued him not to reach too far to avoid loss of balance or falling out of the chair. He reached outside of his base of support multiple times in several movement planes. Given that this participant performed a considerable amount of exercise the day prior, the interventionist chose to have the participant test an adventure game. The participant noted that he was interested in Star Wars. Thus, the interventionist chose to have the participant play the Star Wars game Vader Immortal: an adventure game, with many handheld interactions within the virtual environment and sections of active play with a laser sword. As the game was only available on the Oculus store, WalkinVR software could not be used, and this caused several usability issues during gameplay in the seated position. On several occasions, the game would ask the participant to reach down or forward to interact with an object, a movement that was not achievable while seated, and the patient was not yet strong enough to tolerate dynamic standing activities. To overcome this obstacle, the interventionist had to equip the headset and complete the task and then reequip the headset to the patient. Nevertheless, the patient was so engaged and interested in the gameplay that he did not want to switch to another game. He completed nearly the entire game during the available playtime (maximum of 1 hour due to other appointments). Again, the therapist noted that, before VR gaming, he would never have participated in 50 minutes of physical therapy. The 2 sessions of VR gaming allowed the physical therapist to build rapport with the patient. For the remainder of his hospital stay, he was more willing to participate in progressive strengthening exercises and higher-level mobility activities.

It must be noted that the patient was receiving a blood transfusion via the peripheral intravenous drip line. This line was occluded a couple of times throughout play, sending alerts to the nursing staff, who came in and helped fix the occlusion while the participant continued playing. Physicians also came in to check the status of the patient and asked him several questions, all while the headset was still equipped and the patient was playing. He appeared to enjoy the gameplay, and thus, the physicians did not want to disturb him because he was highly inactive throughout his stay. After completing the session, taking off the headset, and being transferred back to the bed, the patient vomited a few times. The patient had a history of emesis throughout the day due to his underlying pancreatitis. The patient’s mother and hospital staff did not believe that the issue was due to the VR play. The patient also did not report feeling motion sick. It must be noted that this was case 2’s last session because his therapists felt that he was now motivated to engage in physical therapy interventions outside of his room to progress toward his functional baseline.

In summary, case 2 was able to start immediately with active exergaming and long gaming sessions. The sessions encouraged him to be more active in the PICU and provided a motivational boost to engage in physical therapy. Gaming without the adaptive capabilities of the WalkinVR software was noticeably difficult and interruptive. The teamwork between the therapist and nurse was needed to ensure the safety of the treatment by providing line management and stand-by assistance to decrease fall risk, whereas the interventionist was focused on the technical aspects of the equipment and gameplay.

## Discussion

### Principal Findings

This case study was the first to examine the feasibility of using HMD gaming with early mobilization therapy among adolescents in the PICU. A novel component of this study was that it used adaptive software with one of the latest consumer-available gaming HMDs that have replaced the less immersive forms of VR gaming (Nintendo Wii, X-box Kinect, and PlayStation Eye, all of which have been discontinued by their manufacturers). Overall, the findings suggest that HMDs could be delivered by an early mobilization physical therapist and research assistant to pediatric patients. Patients were able to participate in both active and nonactive single-player games. No serious adverse events that appeared directly related to VR gameplay occurred. Nevertheless, adverse events did occur (stomach hotness and vomiting). Thus, the safety of this treatment must be explored in a larger feasibility study. The findings of this study regarding process, resources, and management should assist in informing the development of a feasibility study with a more structured intervention dose and should help in assessing the impact of this dose on a precise health outcome.

For example, moderate exercise is a critical method of improving physical fitness and preventing deconditioning and comorbidities among adolescents with disabilities both within and outside of a formal rehabilitation setting [21-23]. This study demonstrated that adolescents in the PICU could potentially reach moderate intensities of exercise using only their arms for exercise. This finding agrees with the current early mobilization paradigm: early exercise therapy is likely safe and beneficial for pediatric patients [2]. Further research is needed to quantify both physiological responses and adaptations to VR exergaming in the PICU.

Perhaps the greatest benefit of VR gaming was that it motivated the adolescents to become more active, alert, and engaged with their typical physical therapy treatment. Both patients were resting most of the day in the PICU until they received VR treatment. After 2 sessions, the patients could more easily be persuaded to participate in early mobilization physical therapy. The underlying mechanisms of this behavioral change warrant further examination so that this response can be replicated. Moreover, future research should examine the potential effects of VR treatment plus early mobilization therapy on critical psychosocial outcomes in the PICU, such as anxiety, pain, and depression.

### Recommendations for Implementing HMD Gaming in the PICU

#### Process

Process recommendations for implementing VR in the PICU are listed below:

- Prepping the patient for gameplay will require joint support from an early mobility therapist and nurse.
- Begin with an introductory game to VR (use more exploratory games with minimal required activity or movement).
- During the introductory session, adjust the movement boost and location of the controllers within the WalkinVR software while the person is playing the game (record the settings for each game, to quickly input in subsequent sessions).
- Introduce active games (eg, Thrill of the Fight or Beat Saber) once the patient becomes comfortable with the VR environment.
- Provide positive verbal encouragement to enhance motivation.
- Gradually reduce the movement boost of the controllers in WalkinVR as the patient progresses in the game and successfully completes tasks, to increase energy expenditure.
- Sessions should last approximately 45 minutes, with 30 minutes of playtime and 15 minutes for setup and cleanup.

#### Resources

Recommendations for VR gaming resources in the PICU are listed below:

- WalkinVR software is required for playing most games adequately from the bedside.
- The gaming laptop must have adequate processing and graphics power to maximize the frames per second display of the HMD.
- Gaming genres should include active video games, narrative adventure games, recreation or sports, and potentially mindfulness or meditative games.
- Staffing should include supervision by an early mobility therapist and an interventionist and support from nearby nursing staff due to the potential for adverse events, including line or equipment dislodgement, unsafe change in vital signs, or severe patient discomfort.

### Limitations

Inherent within a case study design, study findings should be interpreted with caution and will need to be confirmed in a larger feasibility study. The study only included 2 males, and we are unsure whether females will respond similarly to the treatment. Another limitation was the lack of objective outcomes, which could have provided estimates for treatment effects in an efficacy trial. Moreover, both the participants were experienced with video games. Further research is needed to identify whether VR gaming with HMDs is acceptable in the PICU among nonexperienced gamers. Finally, the narrative report was presented from the lens of the interventionists, not the participants. The adolescent participants did not express a desire to be formally interviewed. This prevented the study from providing a user-centered perspective on usability or feasibility.

### Conclusions

This study demonstrated that incorporating consumer-available VR gaming within the early stage of early mobilization therapy is likely feasible among patients in the PICU. However, further quantitative and qualitative research is needed to confirm the safety, acceptability, and potential benefits of such treatments on scientific outcomes.

## Appendix

Multimedia Appendix 1 Video demonstration of virtual reality with a head-mounted display in the pediatric intensive care unit.

## Abbreviations

HMD: head-mounted display
PICU: pediatric intensive care unit
VR: virtual reality
